# Total proximal tibial osteotomy and cranial closing wedge ostectomy for treating concomitant medial patellar luxation and cranial cruciate ligament disease in dogs with excessive tibial torsion

**DOI:** 10.1371/journal.pone.0327247

**Published:** 2025-07-22

**Authors:** Alefe Luiz Caliani Carrera, Eloy Henrique Pares Curuci, Dayvid Vianêis Farias Lucena, Lucas Vasconcelos Costa, Rodrigo Carvalho de Souza Faustino, Stefânia Carolino Claudino Silva, Luis Gustavo Gosuen Gonçalves Dias, Bruno Watanabe Minto

**Affiliations:** 1 Department of Veterinary Clinics and Surgery, School of Agricultural and Veterinary Studies, São Paulo State University, São Paulo, Brazil; 2 Veterinary Orthopedics, Veterinary Surgeon, São Paulo, Brazil; 3 Department of Veterinary Medicine, Federal University of Jatai – UFJ, Jataí, Goias, Brazil; 4 Department of Zootechnics, State Univerity of Maringá – UEM, Maringá, Parana, Brazil; Yarmouk University, JORDAN

## Abstract

Medial patellar luxation (MPL) and cranial cruciate ligament disease (CCLD) frequently co-occur in dogs, with excessive tibial torsion adding surgical complexity. This study evaluates the clinical outcomes of total proximal tibial osteotomy (tPTO) combined with cranial closing wedge ostectomy (CCWO) for treating MPL and CCLD in dogs exhibiting excessive external tibial torsion. A retrospective case series was conducted at a single referral hospital, including dogs with concomitant MPL and CCLD, exhibiting external tibial torsion ≥20°, and treated with tPTO and CCWO. A total of 31 patients met the inclusion criteria. Postoperatively, tibial torsion was corrected in all cases, with 74.2% (n = 23) achieving near 0° alignment. The tibial plateau angle decreased from 23.0° ± 5.0° to 7.0° ± 3.4°. Antirotational techniques were used to prevent reluxation in 25.8% (n = 8) of cases because of stifle soft tissue laxity. The initial success rate for patellar and stifle stability was 87.1%, whereas 12.9% (n = 4) of cases exhibited patellar reluxation, necessitating surgical reintervention with antirotational techniques. Bone healing averaged 70.2 ± 21.2 days, aligning with full clinical recovery. Following the four surgical reinterventions, all patients achieved patellar and stifle dynamic stability with normal weight bearing. The combination of tPTO and CCWO proved effective for treating concomitant MPL and CCLD in dogs with excessive tibial torsion, demonstrating favorable outcomes and complication rates. This technique should be considered for managing these complex cases.

## Introduction

Medial patellar luxation (MPL) and cranial cruciate ligament disease (CCLD) are common stifle conditions in dogs [1]. These conditions coexist in approximately 25% of MPL cases, highlighting the need for effective simultaneous treatment [2]. The exact association between MPL and CCLD remains unclear; however, MPL may predispose dogs to CCLD [3].

Surgical correction of MPL with concurrent CCLD aims to level the tibial plateau angle (TPA) and realign the stifle extensor mechanism (SEM) [4]. Traditional approaches include modified tibial plateau leveling osteotomy (mTPLO) [5] or TPLO combined with tibial tuberosity transposition (TTT) [6]. However, these methods have limitations in terms of efficacy and safety in cases of excessive tibial torsion (> 20°) [5,6]. Additionally, TPLO with TTT introduces higher mechanical stress owing to multiple osteotomies and additional implants [6]. Conversely, cranial closing wedge ostectomy (CCWO) is recommended for the treatment of CCLD because of its efficacy in reducing the TPA [7]. However, this may not be effective in restoring SEM alignment.

External tibial torsion is closely associated with MPL [8]. Many cases may be addressed with the standard TTT technique [9]. However, when the tibial torsion exceeds approximately 20°, correction becomes more challenging. Tuberosity transposition alone is neither safe nor effective in such cases, leading to a higher risk of complications and patellar reluxations [10,11].

This study evaluates the clinical outcomes of total proximal tibial osteotomy (tPTO) combined with CCWO for dogs with concurrent CCLD, MPL, and excessive external tibial torsion. We hypothesize that performing both procedures at the same osteotomy site enables simultaneous TPA leveling and SEM realignment through proximal tibia detorsion. While the clinical application of this approach appears promising, no previous study has provided a detailed description of the technique nor has assessed its efficacy.

## Materials and methods

### Study design and inclusion criteria

This study was approved by the Ethics Committee for Animal Use of the São Paulo State University (protocol number: 08498/2023). Considering that this was a retrospective analysis, informed consent from the owners of the patients was not required. This retrospective study was conducted between 2019 and 2023, and the data were accessed on January 12, 2024. All procedures were performed at a single referral hospital by the same surgeon and involved canine patients who underwent tPTO in combination with CCWO as surgical treatments for concurrent CCLD and MPL with excessive external tibial torsion (≥ 20°) in the same limb.

### Medical record search and data collection

Patient data were retrieved from the hospital database, including breed, age, weight, patellar luxation grade [12], TPA, femoral and tibial deformities (measured using radiography or computed tomography – CT), surgical procedures, complications during and after surgery, and follow-up period.

### Bone angle measurement and deformities

**Fig 1:**
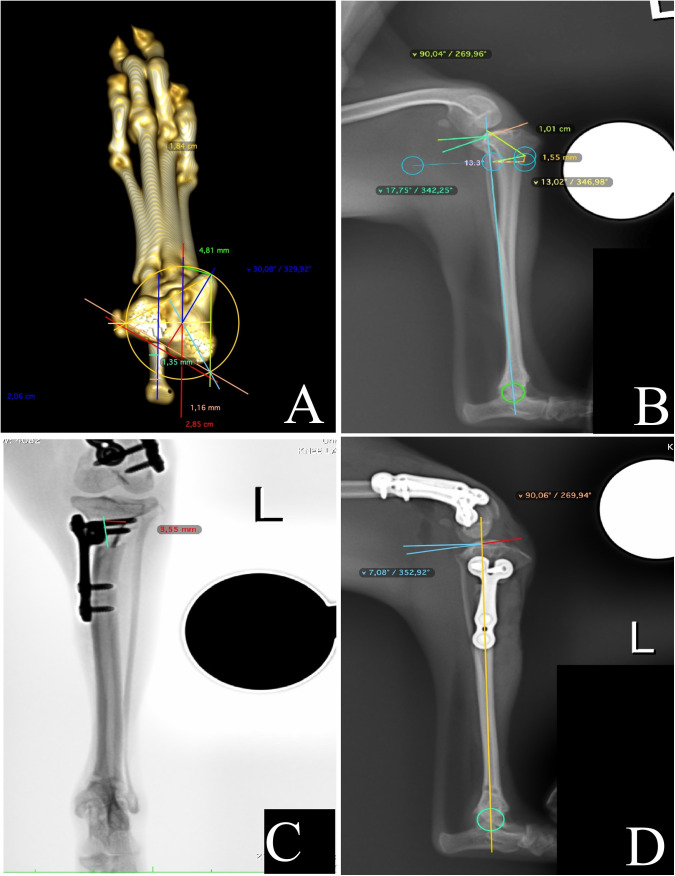
Preoperative preparation for patients diagnosed with both medial patellar luxation and cranial cruciate ligament disease who underwent proximal total tibial osteotomy and cranial closing wedge ostectomy. Objective tomographic measurement of tibial torsion (A); radiographic planning with the tibial plateau angle (TPA) and wedge measurements (B); subjective radiographic measurement of postoperative tibial torsion (C); postoperative TPA measurement (D).

Tibial torsion was preferably measured using three-dimensional CT. A cross section of the stifle was taken, highlighting the proximal region with the tibial plateau, the caudal view with the calcaneus in the distal portion, and the cranial view with the metatarsus. The tibial crest was located in the cranioproximal region (Fig 1A). Precise measurements were obtained using OsiriX (Pixmeo SARL, Bern, Switzerland) and VPop Pro (VETSOS Education Ltd., Shrewsbury, United Kingdom), according to a previous study [13]. When objective measurement was not feasible, a subjective analysis was performed using craniocaudal or caudocranial projections as a reference in 5° steps [14]. Patients eligible for either objective or subjective analysis were required to have tibial torsion ≥ 20°. The TPA was evaluated using VPop Pro (Fig 1B).

Femoral deviations were assessed using established reference angles [15]. Cases with distal femoral varus ≥ 8° (anatomical lateral distal femoral angle - aLDFA ≥ 94° [14]) underwent femoral osteotomy per previously described methods [16]. The patellar groove depth was measured intraoperatively or via CT scanning, with grooves < 50% of patellar height classified as shallow and undergoing deepening procedures.

Postoperatively, the TPA, SEM alignment, residual tibial torsion, and bone healing progression were assessed. Tibial torsion was evaluated subjectively [14] (Fig 1C), whereas the postoperative TPA was measured using VPop Pro (Fig 1D).

### Surgical treatment

**Fig 2:**
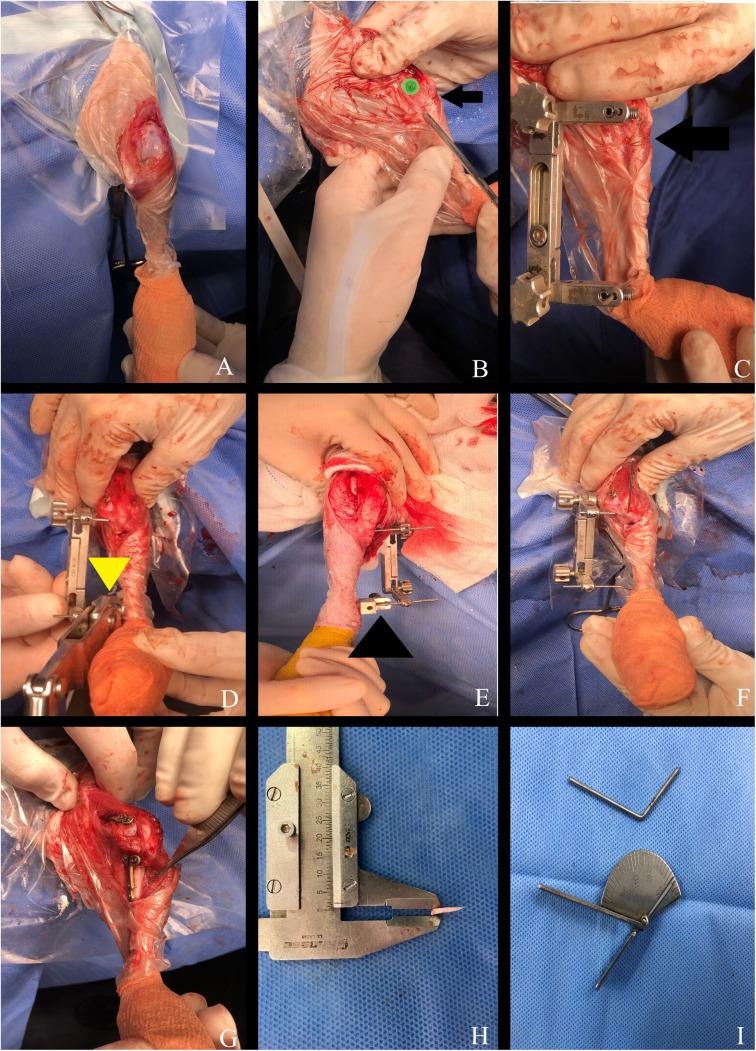
Intraoperative procedures of proximal total tibial osteotomy combined with cranial closing wedge ostectomy in patients with concomitant medial patellar luxation and cranial cruciate ligament disease. Craniomedial proximal tibial surgical access, with lateral arthrotomy, showing external tibial torsion (A). Stifle joint center demarcation with a needle and tibial crest as a reference for the osteotomy baseline, perpendicular to the sagittal plane of the tibia (arrow) (B). Visualization of the jig following implantation and demarcation of the wedge base (arrow) (C). After the ostectomy, the distal wire of the jig was twisted with a plate bender (yellow arrowhead) (D). Wire bender (black arrowhead) at the distal wire of the jig as an alternative for detorsion (E). After the stifle extensor mechanism realignment, the jig was locked and a Kirschner wire was inserted 45° to the osteotomy line (F). The osteotomy line was reduced, and a locked plate and screws were inserted (G). Intraoperative measurement and confirmation of the removed wedge (H). Intraoperative measurement and reassessment of tibial detorsion through the distal wire using a goniometer (I).

**Fig 3:**
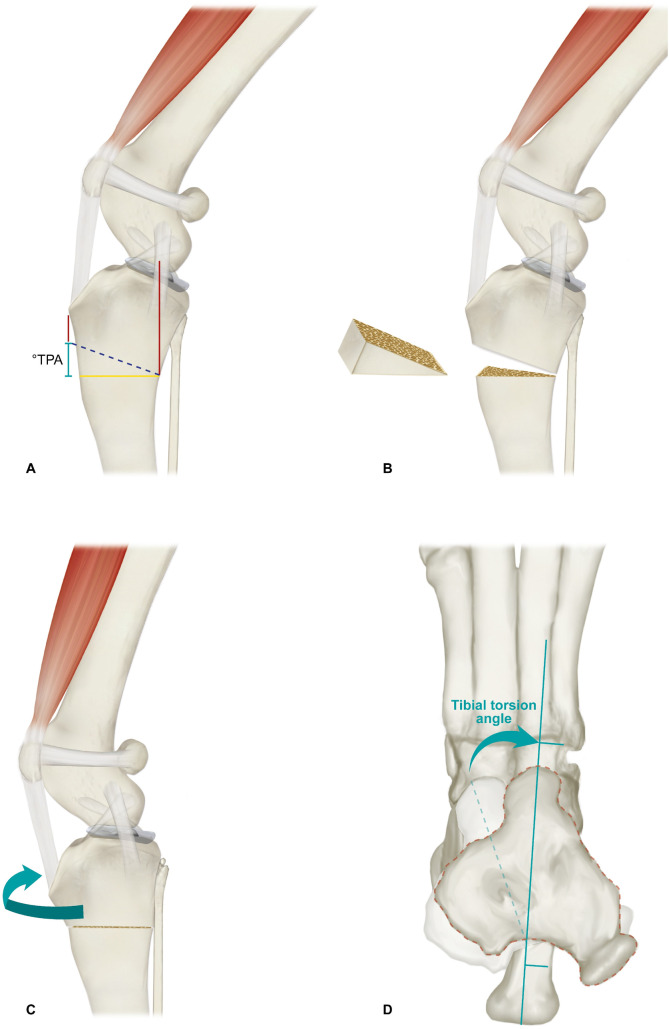
Diagram illustrating the combination of proximal total tibial osteotomy and cranial closing wedge ostectomy in dogs. Red lines indicate the measurements for key anatomical landmarks used to demarcate the osteotomy lines, including the stifle joint center and the insertion point of the patellar ligament at the tibial crest. The opening angle of the wedge was similar to the tibial plateau angle (A). Removal of the wedge following the completion of osteotomies for both lines (B). After the ostectomy, the proximal and distal tibia segments were realigned, resulting in a perpendicular osteotomy line relative to the sagittal tibial plane, causing lateral rotation of the proximal portion (C). Proximodistal view demonstrating the proximal portion rotation until alignment with the talus, using the calcaneus and the fourth tarsal bone as reference points, with the rotation angle being similar to the previous tibial torsion (D).

TPA correction was achieved using CCWO [17], with a slightly more proximal osteotomy [18] (Figs 2A,2B, and 3A), targeting a final TPA of approximately 8° [18]. The SEM alignment was evaluated intraoperatively using anatomical reference points: proximal (patellar ligament insertion and patellar position) and distal (center point between the talus and calcaneus) [13,14]. The ultimate objective was to realign these points using tPTO.

After establishing the first osteotomy line, oriented perpendicularly to the sagittal axis of the tibia (Fig 3B), this line served as the base for the subsequent construction of the wedge (Fig 3C) distal to the insertion point of the patellar ligament. The jig was inserted with a proximal wire cranial to the collateral medial ligament of the stifle (Fig 2C). To achieve the necessary proximal rotation, a plate bender/bender device was used to bend the distal wire of the jig to the cranial direction (Fig 2D,2E). Subsequently, osteotomies were performed and the bone wedge was removed (Fig 3B). After ostectomy, bone reduction and tibial realignment were performed (Fig 3C). Fragment contact was achieved by aligning the caudal cortex of the tibia, as aligning the cranial cortex was not feasible because of the detorsional movement.

Bending the distal jig wire allowed proximal tibial rotation without removing the jig (Figs 2D and 3D). Proximal tibial adjustment was performed until the SEM was aligned. In patients who underwent radiographic assessment, this alignment was determined subjectively, whereas in those who underwent tomographic evaluation, it was assessed objectively based on preoperatively measured tibial torsion. Following the realignment of the limb, the osteotomy line was temporarily stabilized with a wire, which was later removed once final stabilization was achieved using locking plates and screws (Fig 2F,2G).

Intraoperative assessment of CCWO was performed by measuring the dimensions of the removed wedge (Fig 2H), whereas that of tPTO was performed by measuring the torsion in the distal wire using a goniometer (Fig 2I) or by measuring the millimeters of tibial crest translation compared with the original point. During the intraoperative evaluation, any residual tibial rotation relative to the distal femur was assessed. If excessive tibial rotation was observed, preventive antirotational techniques, such as lateral fabellotibial suture or elastic external skeletal fixation [19–21] were applied until joint stabilization was achieved.

### Data analysis

Data organization and tabulation were performed using Microsoft Excel version 2021 (Microsoft Corporation, Redmond, WA, USA). Statistical analyses were conducted using R software (R Core Team, Vienna, Austria; version 4.3.3, 2023) with the *stats* and *rcompanion* packages. Quantitative parameters were subjected to statistical analysis. Differences in quantitative variables were evaluated using Student’s t-test and Pearson’s correlation with a 95% confidence interval. Qualitative variables were evaluated using descriptive analyses and presented as absolute and relative (%) values. McNemar’s test and Spearman’s rank correlation coefficient were used for specific analyses.

## Results

### Patient inclusion

In total, 31 dogs met the inclusion criteria. Toy breeds were predominant, with Pomeranian being the most common (54.5%, n = 20), followed by Yorkshire Terrier, Maltese, Cavalier King Charles Spaniel, and Mongrel (each 5.4%, n = 2). Boston Terriers, Italian Greyhounds, and Poodles each comprised 3.2% (n = 1). The mean age of the patients was 3.0 ± 2.3 years, and the average weight was 4.9 ± 2.8 kg. Most patients had grade III MPL (45.2%, n = 14), followed by grade IV (41.9%, n = 13) and grade II (12.9%, n = 4). The right pelvic limb was the most affected (58.1%, n = 18).

### Preoperative bone angle measurements and deformities

Regarding measurements of bone deviations, radiographic evaluations were performed on all patients, while 64.5% (n = 20) also underwent CT. For tibial deviations, the mean objective external torsion measured via CT was 26.9° ± 9.9°, and the translation yielded a value of 5.5 ± 1.3 cm. The preoperative TPA was 23.0° ± 5.0°. Concurrent femoral deformities were detected in 51.6% (n = 16) of the patients, with femoral varus being the most prevalent type, accounting for 93.7% (n = 15), with a mean of 11.1° ± 5.4°, considering the anatomical lateral distal femoral angle as 94°. Surgical correction was performed in all patients with femoral deviation (51.6%, n = 16) using distal femoral osteotomy.

### Surgical treatment

Trochleoplasty was performed in 80.3% (n = 25) of patients, with the wedge-shaped cutting technique most frequently used (74.2%, n = 23). During surgery, tibial rotation was observed in 25.8% (n = 8) of cases, leading to patellar laxity. To address this, antirotational techniques were used, with sutures being the most common method used (19.3%, n = 6), and external fixation used in 6.4% (n = 2) of cases. None of these eight patients experienced postoperative patellar reluxation.

### Postoperative bone angle measurements and deformities

The postoperative TPA decreased to 7.0° ± 3.4°, which was significantly lower than that observed before surgery (Table 1). Additionally, the technique was effective regardless of the preoperative TPA according to Pearson’s correlation analysis (R = 0.02).

**Table 1:** Statistical correlation between the pre- and postoperative tibial plateau angle in dogs with medial patellar luxation and cranial cruciate ligament disease undergoing proximal total tibial osteotomy and cranial closing wedge ostectomy.

Tibial plateau angle					
	Mean	Standard error	Standard deviation	Variation coefficient	p Value
Before surgery	23.02	0.91	5.03	0.21	
After surgery	7.06	0.63	3.47	0.49	0.02^*^

Statistical *p*-value of < 0.05 indicates a significant difference as determined using Student’s t-test (^*^).

Postoperative evaluation of detorsional tibial osteotomy revealed that 45.2% (n = 14) of the patients exhibited tibial torsion within the range of 0°–5°, as determined subjectively. Subjective internal tibial torsion was observed in 41.9% (n = 13) of patients, with 29.0% (n = 9) falling within the 0°–5° range and 12.9% (n = 4) within the 5°–10° range. None of the patients with iatrogenic internal tibial torsion experienced complications such as lateral patellar luxation. In total, 74.2% (n = 23) of the patients were considered to have tibial torsion near 0°, considering a threshold of 5° for internal or external torsion, leading to satisfactory SEM realignment.

### Outcomes

Postoperative complications were observed in 16.1% (n = 5) of the patients owing to patellar reluxation (12.9%, n = 4) and iatrogenic tibial valgus of 5° (3.2%, n = 1). All four patients with patellar reluxation underwent surgical reintervention. Antirotational techniques had not been used during the initial surgery for these patients. Following reintervention with antirotational techniques, all cases were successfully resolved. McNemar’s test did not reveal a significant difference between postoperative complications and the use of intraoperative antirotational techniques (*p*-value = 1). The initial success rate for stifle stability was 87.1%, accounting for patients who experienced reluxation. After re-intervention, it increased to 100%, with no occurrence of reluxation or positive cranial tibial thrust test results.

The follow-up period extended until complete radiographic bone healing was achieved, with a mean duration of 70.2 ± 21.2 days for bone healing. No other complications were identified during this period.

### Data analysis

The results of the Spearman’s correlation analysis of the parameters examined are shown in Table 2. A significantly strong positive correlation was observed between wedge height, body weight, and the preoperative TPA and between bone healing time and preoperative tibial torsion, indicating that these factors tended to increase together. Additionally, a significant moderate correlation was found between the preoperative TPA and body weight. Conversely, moderate negative correlations were observed for certain variables, indicating an inversely proportional relationship. These factors include age, torsion, the postoperative TPA, and body weight.

**Table 2:** Spearman’s rank correlation coefficient results for different variables in dogs diagnosed with medial patellar luxation and cranial cruciate ligament disease and treated with proximal total tibial osteotomy and cranial closing wedge ostectomy.

	Age	Weight	Tibial torsion	Preoperative TPA^a^	Wedge height (CCWO^b^)	Postoperative TPA^a^	Bone healing time	p-Value
Age	1.00							
Weight	0.10	1.00						
Tibial torsion	−0.37*	0.06	1.00					0.04
Preoperative TPA^a^	0.11	0.36*	0.06	1.00				0.04
Wedge height (CCWO^b^)	0.33	0.73*	0.12	0.73*	1.00			< 0.01
Postoperative TPA^a^	−0.32	−0.45*	0.32	0.02	−0.29	1.00		0.01
Bone healing time	0.26	−0.06	0.61*	−0.07	−0.01	0.14	1.00	< 0.01

Positive results indicate a positive correlation, whereas negative results indicate a negative or contradictory correlation.

^a^Tibial plateau angle.

^b^Cranial closing wedge ostectomy.

*Statistical significance (p < 0,05)

## Discussion

Given the frequent coexistence of MPL and CCLD, both requiring surgical intervention [5], the combination of tPTO and CCWO presents a viable treatment option. Previous studies have explored surgical approaches for these conditions [6]; however, limitations such as complication rates and the effectiveness of correcting excessive tibial torsion have been noted [6,11]. The findings of this study confirm that tPTO is effective in correcting transverse-plane tibial deformities and that CCWO successfully neutralizes the TPA, addressing CCLD. Rapid bone healing and satisfactory complication rates further support the efficacy of these techniques. These results align with the hypotheses proposed by the authors and are consistent with other findings on the use of osteotomies to neutralize the TPA [20–22], reinforcing the clinical utility of combining tPTO and CCWO in correcting excessive tibial torsion.

Traditionally, patients with tibial torsion of up to 20° have been treated with TTT [10,11]. However, studies addressing excessive torsion are limited. Consequently, comprehensive analyses of transverse osteotomy of the tibia and proximal tibial realignment are required. As evidence of the efficacy of the combined approach, the results of this study revealed that all patients demonstrated a reduction in external tibial torsion postoperatively, with 74% achieving subjective postoperative torsion levels close to 0°, considered the ideal point for SEM alignment. Additionally, it allowed for the simultaneous management of CCLD at the same osteotomy site using CCWO. CCWO has been demonstrated to effectively reduce and neutralize the TPA [22], as indicated by the significant difference between the pre- and postoperative TPA. These findings align with existing literature, reinforcing tPTO + CCWO as a viable alternative for treating MPL with concurrent CCLD and tibial torsion.

These findings also demonstrate that CCLD does not necessarily contraindicate detorsional tibial osteotomy with tPTO. While TPLO is typically the primary surgical technique for CCLD management [23,24], when MPL is present, a modified TPLO approach involving lateral dislocation of the tibial crest is recommended [25]. However, this approach fails to correct tibial torsion exceeding 20°, a challenge shared with traditional TTT techniques [10,11,25]. Therefore, for cases involving MPL and CCLD with tibial torsion exceeding 20°, this study recommends the use of tPTO + CCWO, which demonstrated a high success rate and favorable recovery outcome.

CCWO, as a stand-alone technique, has demonstrated the ability to effectively reduce the TPA while maintaining low rates of major and minor complications, showing rapid recovery and favorable outcomes within a short period [26]. These findings are consistent with those of the present study, which also identified low rates of major complications and a postoperative TPA at 7°, a classification considered desirable for achieving dynamic stifle stabilization. Furthermore, CCWO may outperform TPLO in addressing an excessive TPA (eTPA) and caudal bowing of the proximal tibia [27], further supporting the use of CCWO when an eTPA is detected concurrently with MPL and when tibial torsion exceeds 20°.

Postoperative complications were observed in 12.9% of cases, indicating a lower complication rate compared with traditional techniques used for treating tibial torsion. For instance, the use of TTT in MPL treatment has been associated with a 25% occurrence of major complications [28], whereas the mTPLO approach resulted in 18.4% [5]. Therefore, tPTO + CCWO exhibited a more favorable safety profile than the established techniques for addressing tibial torsion, irrespective of the presence of CCLD. However, the most prevalent complication in this study was patellar reluxation, which exhibited a higher incidence than that reported in previous MPL treatment studies [9,28]. This increased patellar reluxation may be attributed to residual tibial rotation, possibly resulting from laxity in the periarticular soft tissues, suggesting that tPTO alone may not fully address soft tissue laxity. In addition to tibial torsion, tibial rotation plays a significant role in the pathophysiology of MPL and may be present in certain cases [29]. Therefore, the adoption of antirotational techniques may be necessary when laxity exceeds expectations [19]. For instance, antirotational sutures can reduce the internal rotation of the proximal tibia, thereby alleviating strain on the collateral ligaments and joint capsule [19]. These considerations guided the decision to employ antirotational sutures in some patients in this study, particularly when residual tibial rotation was identified during the perioperative period. Another critical aspect related to internal tibial rotation is its association with rupture of the cranial cruciate ligament, which aids in preventing internal tibial rotation, and its rupture can lead to an increased degree of rotation [30]. Consequently, when CCLD and MPL coexist, the implementation of antirotational techniques is highly recommended.

This study has confirmed the effectiveness of tPTO in reducing tibial torsion and CCWO in neutralizing the TPA; however, further research is needed to evaluate limb function biomechanically. The use of radiographic evaluation for some patients, instead of more accurate tomographic imaging [13], may be a limitation in measuring tibial torsion and could be associated with suboptimal postoperative results.

In summary, this study demonstrated the feasibility of performing both surgical techniques at the same osteotomy site. tPTO successfully reduced tibial torsion in 100% and realigned the limbs in 74% of patients. CCWO effectively reduced the TPA to the expected range for stifle dynamic stabilization in all cases. The main complication observed was patellar reluxation, probably due to laxity of the periarticular soft tissues. Residual tibial rotation served as an important criterion for considering the use of antirotational techniques during surgery or for addressing postoperative complications. These findings suggest that tPTO + CCWO is a highly effective and safe alternative for managing concurrent MPL and CCLD in dogs with excessive external tibial torsion, offering promising clinical outcomes.

## Conclusion

The combination of tPTO and CCWO proved to be an effective surgical approach for addressing concurrent MPL and CCLD in dogs with excessive external tibial torsion, with lower complication rates compared with those observed when using conventional techniques.

## Supporting information

S1 File Supporting information and dataset containing information on all 31 patients included in the study. (XLSX)
