# Correction: Total proximal tibial osteotomy and cranial closing wedge ostectomy for treating concomitant medial patellar luxation and cranial cruciate ligament disease in dogs with excessive tibial torsion

**DOI:** 10.1371/journal.pone.0335371

**Published:** 2025-10-23

**Authors:** Alefe Luiz Caliani Carrera, Eloy Henrique Pares Curuci, Dayvid Vianêis Farias Lucena, Lucas Vasconcelos Costa, Rodrigo Carvalho de Souza Faustino, Stefânia Carolino Claudino Silva, Luis Gustavo Gosuen, Gonçalves Dias, Bruno Watanabe Minto

The Funding statement for this article is incorrect. The correct Funding statement is as follows: This study was financed in part by the Office for Graduate Programs of São Paulo State University (PROPG/UNESP) through call nº 23/2025.
